# Systematic Distortions in Vertical Placement of Features in Drawings of Faces and Houses

**DOI:** 10.1177/2041669517691055

**Published:** 2017-01-01

**Authors:** Neil R. Harrison, Julia Jones, Simon J. Davies

**Affiliations:** Liverpool Hope University, UK

**Keywords:** attention, attention to features/objects, face perception, long-term memory

## Abstract

A crucial part of accurately drawing portraits is the correct vertical positioning of the eyes. Non-experts typically place the eyes higher on the head than they are actually located; however, the explanation for this remains unclear. In Experiment 1, participants drew faces from memory and directly copied from a photograph, to confirm whether biases in observational drawings were related to biases in memory-based drawings. In Experiment 2, participants drew a cat’s face, to test explanations by Carbon and Wirth for the positional bias: the ‘view-from-below, the ‘head-as-box’, and the ‘hair-as-hat’ explanations. Results indicated that none of these three explanations could fully account for the vertical positioning biases observed in drawings of the cat’s face. The findings are discussed in relation to the idea that distortions of vertical alignment in drawings may be related to the position of the most salient features within a face or object.

## Introduction

Accurate drawing requires the skillful co-ordination of perceptual, motor, and decision-making processes, and consequently most people are rather poor at producing accurate depictions of objects (for review, see Chamberlain & Wagemans, 2016). To achieve an accurate depiction of an object, a drawing must reproduce the correct relative spatial positions of the object’s features. This is particularly important in portrait drawing where the correct spatial arrangement of the facial features influences the accuracy of face recognition (Rotshtein, Geng, Driver, & Dolan, 2007). One of the most important spatial relationships in portraiture is the vertical positioning of the eyes on the head. Despite this central importance, it was empirically demonstrated by Carbon and Wirth (2014) that adults typically place the eyes too high up the head, and indeed subsequent research showed that the degree of recognisability of drawn faces is lower for faces containing larger errors in vertical eye placement (Ostrofsky, Cohen, & Kozbelt, 2014).

The reason people tend to draw the eyes too high up the head is still poorly understood. There are multiple potential sources of errors in drawings (Chamberlain & Wagemans, 2016), but of particular relevance here is a class of drawing errors called ‘negative categorical schemas’ (Chamberlain & Wagemans, 2016), which cause drawings to be influenced more by internal representations of the model than by the actual model itself (see also Matthews & Adams, 2008; Picard & Durand, 2005). One such negative categorical schema is the long-term memory (LTM) representation of the to-be-drawn object, which can interfere with and distort the accuracy of its depiction. Indeed, it has been shown that errors in vertical positioning of the eyes on the head are associated with errors of vertical eye position in graphic LTM representations of the human face (Ostrofsky, 2015). As noted by Ostrofsky (2015), the nature of the graphic LTMs is currently unknown but could potentially include visual representations of previously seen faces, procedural information about drawing faces, or declarative knowledge about rules governing the placement of facial features. In Experiment 1 we aimed to replicate the results of Ostrofsky (2015), by asking participants to first perform a free drawing of a face (i.e., a memory-based drawing, without a model). Accuracy of the memory-based drawing was compared with the accuracy of a subsequently produced observational drawing of a face based on a photograph. Associations between errors in the memory-based and the observational drawings would provide further evidence of the influence of graphic LTMs on the production of accurate depictions of a model.

Nevertheless, our understanding of the origins of the distorted representation of the vertical positioning of the eyes on the head remains incomplete. Carbon and Wirth (2014) put forward three potential explanations. According to the ‘face-from-below’ explanation, the perspective from which children typically view human faces (i.e., from below) could distort subsequent mental representations of facial feature configurations (see also Wirth & Carbon, 2010). Here we wanted to test this ‘extreme perspective’ explanation in Experiment 2, by asking participants to draw a face belonging to a species (a cat) with similar feature configurations (i.e., two eyes above a nose and mouth), but that is customarily viewed by children and adults from above due to its small size. Accordingly, if the ‘extreme perspective’ account of vertical eye-drawing errors is correct, the bias to position the eyes too far up the head should be absent (or even reversed) when drawing the cat’s face.

A second explanation proposed by Carbon and Wirth (2014) for the distortion in eye placement was the ‘hair-as-hat’ explanation, in which the hair is not viewed as belonging to the head, and therefore, the eyes are positioned further up the face. If the ‘hair-as-hat’ explanation is correct, the eye-position bias should be absent for drawings of the cat’s face, as the cat’s hair is distributed across the face, rather than being located on the top of the head, as with humans. A third explanation put forward by Carbon and Wirth (2014) was the ‘head-as-box’ explanation, where the convexity of the forehead is not taken into consideration, so the top of the head is represented lower in the drawn depiction. Instead of showing a convexity, the outline of the top of the cat’s head to be depicted in Experiment 2 is overall concave (see Figure 3(a)), due to the position of the inner edges of the ears (in other words, the outline of the top of the cat’s head including the ears forms a ‘U’ shape). Therefore, we would expect the bias to be reduced or eliminated for the drawing of a cat’s head if the ‘head-as-box’ explanation is correct, as the top of the head should no longer be represented lower, since the outline of the top of the cat’s head is not convex.

Lastly, we tested whether a systematic upward positioning bias of object features is specific to faces, or whether such a bias generalises to non-face objects (a house).

## Experiment 1

### Methods

#### Participants

Twenty-seven participants took part in the experiment. The mean age was 31.7 years (*SD* = 10.7); 19 were females, and all had normal or corrected-to-normal vision. All participants reported that they considered themselves novices at drawing. Eleven participants reported formal training in drawing (at school). The experiment was approved by the Ethics Committee of the Psychology Department at Liverpool Hope University.

#### Materials

**Figure 1.**
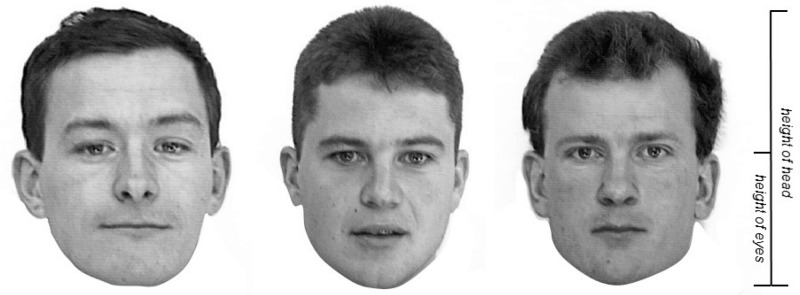
Faces used as stimuli for the observational drawing task. Permission to reproduce the images was provided by the administrator of the Psychological Image Collection at Stirling. The eye height ratio was calculated by dividing the height of the eyes by the height of the head (right).

In the memory-based drawing task, participants were given a white A4 sheet of paper with a box measuring 100 mm (length) × 75 mm (width) in which to draw a face from memory. In the observation-based drawing task, a face stimulus was presented in the upper half of a separate sheet of paper. Each participant copied one of three male faces in frontal view with neutral expressions taken from the ‘Aberdeen set’ from the Psychological Image Collection at Stirling (pics.stir.ac.uk; see Figure 1). The faces were modified by removing the area under the jaw and converting to greyscale. The faces were approximately 90 mm in length, positioned in a box of 100 mm (length) × 75 mm (width). Participants were instructed to draw their depictions inside a box (100 mm × 75 mm) positioned in the lower half of the sheet. Participants were given a 0.5-mm HB mechanical pencil and eraser.

#### Procedure

Participants performed a memory-based drawing task, an observation-based drawing task, and then completed a questionnaire about drawing expertise. The order was the same for all participants (cf. Ostrofsky, 2015), and participants were asked to make their pictures as accurate and realistic as possible. Participants had up to 5 minutes to complete each drawing.

##### Memory-based drawing task

Participants were instructed to draw a picture of a human face, viewed from the front, and to include at least the following features: eyes, nose, mouth, ears, hair, jaw, and neck.

##### Observation-based drawing

Participants copied one of the three faces shown in Figure 1 (each face was copied by nine participants).

##### Drawing questionnaire

Participants were given a two-item questionnaire to measure their self-reported drawing skill. The first item asked whether the participant had ever received formal instruction or tuition in painting or drawing. The second item asked whether they considered themselves to be a novice or an expert at drawing.

##### Measurement of eye position error

Following Carbon and Wirth (2014), we first calculated the ratio of the distance between the position of the tear duct (*endocanthion*) and the tip of the chin (*gnathion*) divided by the distance between the highest point of the head (*vertex*) and the *gnathion*. For the memory-based drawing task, we statistically compared the ratio obtained from each participant’s drawing with the mean eye level ratio given in craniometric studies (i.e., .477, see Farkas, Hreczko, & Katic, 1994), and for the reproduction task, we compared each participant’s ratio calculated from their drawing with the ratio derived from the associated stimulus face.

### Results and Discussion

#### Memory-based drawing task

**Figure 2.**
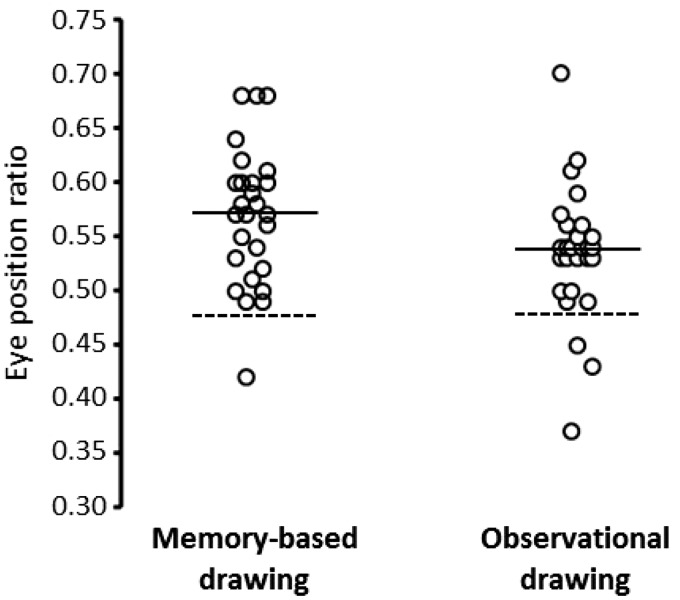
Eye position ratios are plotted for each participant in the memory-based drawing task and the observational drawing task. For the memory-based task, the dashed horizontal line shows the eye position ratio determined from craniometrics studies. For the observational task, the dashed horizontal line indicates the mean eye position ratio of the to-be-depicted face. In both plots, the solid horizontal line displays the mean eye position ratio of the drawn faces.

The eye position ratios for memory-based drawings are plotted for each participant in Figure 2*.* A one sample *t* test revealed a significant difference, *t*(26) = 7.35, *p* < .001, Cohen’s *d* = 1.55, where the value of the eye position ratio was higher for the participants’ drawings (*M* = .57, *SD* = .06) compared to craniometric data (*M* = .477).

**Figure 3.**
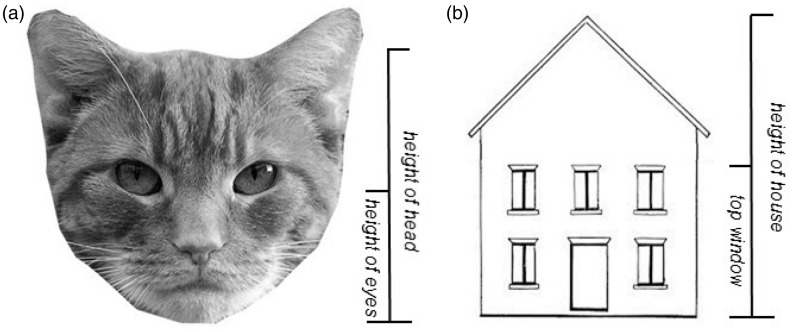
(a) The cat’s face used in the first observational drawing task in Experiment 2. Permission to reproduce this image was provided by iStock. (b) The house participants copied in the second observational drawing task. The measurements used to calculate the vertical position ratios are shown to the right of each figure.

#### Observational task

Individual eye position ratios for observational drawings are displayed in Figure 2. A paired samples *t* test revealed the eye ratio of the participants’ drawing was higher (*M* = .54, *SD* = .06) than the eye ratio of the to-be-depicted faces (*M* = .48, *SD* = .02), *t*(26) = 4.958, *p* < .001, Cohen’s *d* = 1.24.

#### Relationship between observation- and memory-based drawing errors

We tested whether there was an association for the eye position ratio between the memory-based and the observational drawing task. A Pearson’s correlation analysis revealed a significant positive relationship between the ratios in the two tasks (*r* = .434, *p* = .024).

#### Relative eye area analysis

Here we estimated the relative area of the eyes in proportion to the area of the head using the Digimizer 4 software (http://www.digimizer.com) on electronic copies of the drawings, to compare whether the eyes occupied more space (relative to the size of the head) in the drawings compared to the reference photos. The relative area occupied by the eyes in the drawings (*M* = 2.16%, *SD* = .58) was significantly larger than the relative area of the eyes in the to-be-depicted photos (*M* = 1.05%, *SD* = .08), *t*(25) = 9.842, *p* < .001, Cohen’s *d* = 2.12. However, there was no association between relative eye size error (difference between drawn eye area vs. reference photo) and vertical eye position error (*r* = .127, *p* = .536).

#### Additional analyses

We tested whether the eye-position errors were reduced in those participants who reported having received formal training in drawing. In both the memory-based and observational tasks, there were no differences in the magnitude of errors between the two groups (all *p*s > .29). We also tested (using a one-way ANOVA) whether the observational drawing ratio lay between the actual eye height ratio and the LTM ratio. We found a significant difference between the observational drawing ratio, the actual eye height ratio, and the LTM ratios (*F*(2,52) = 29.83, *p* < .001), and post-hoc *t* tests showed that the observational drawing ratio was significantly lower than the LTM ratio (*p* = .013) but was higher than the actual ratio ( *p* < .001).

In summary, the results from Experiment 1 showed that, in both memory-based and observational drawings, participants positioned the eyes too far up the head, replicating the results of previous studies (Carbon & Wirth, 2014; Ostrofsky, 2015). The results also replicated the finding that the spatial positioning error in the observational drawing task was positively correlated with the positional error in the memory-based drawing (Ostrofsky, 2015), providing further support to the theory that graphical representations stored in LTM can influence the accuracy of observational drawings. We also found that that self-reported previous training did not reduce the magnitude of the eye positing errors, and that the eyes were depicted larger in the drawings compared to the reference photos.

In the next experiment, we tested three explanations proposed by Carbon and Wirth (2014) about the origin of the systematic bias proposed (the ‘face-from-below’, the ‘hair-as-hat’, and the ‘head-as-box’ explanations), by asking participants to produce a memory-based and an observational drawing of a cat’s face. We also investigated whether the systematic upward positional bias generalised to the placement of features within a non-face object which displayed a rather different spatial arrangement of features compared to faces (i.e., a house; see Figure 3(b)).

## Experiment 2

### Methods

#### Participants

Twenty-two participants took part in the experiment. The mean age was 23.9 years (*SD* = 8.8); 17 were females, and all had normal or corrected-to-normal vision. All participants reported that they considered themselves novices at drawing. Fourteen participants reported formal training in drawing (at school). Data from one participant in the house drawing tasks were removed due to failure to follow task instructions. The experiment was approved by the Ethics Committee of the Psychology Department at Liverpool Hope University.

#### Materials

The materials were the same as described in Experiment 1, except for the following details: In the observational drawing of a cat, each participant copied the stimulus shown in Figure 3(a), depicting a cat’s face viewed from the front (from iStock (www.istockphoto.com) – item 140272627). The area lying beneath the jaw was removed and the image was converted to greyscale. For the observational drawing of a house, each participant copied a simplified image of a house, where the top of the upper windows was positioned halfway up the house (Figure 3(b)).

#### Procedure

Participants performed a memory-based drawing of a cat’s head, an observational drawing of a cat’s head, a memory-based drawing of a house, an observational drawing of a house, and then completed a questionnaire about drawing expertise. Instructions were the same as for Experiment 1.

##### Memory-based drawing of a cat

Participants were instructed to draw a picture of a cat’s face from memory, as if viewed from the front. Participants were asked to include at least all of the following features: outline of head, eyes, nose, mouth, and ears.

##### Observation-based drawing of a cat

Participants were instructed to produce a copy of the cat’s head shown in Figure 3(a).

##### Memory-based drawing of a house

Participants were instructed to draw a picture of a house, from a frontal viewpoint. Participants were asked to include the following: outline of house, windows, door, and roof.

##### Observation-based drawing of a house

Participants were instructed to produce an accurate copy of the house shown in Figure 3(b).

##### Calculation of drawing errors of cat

To estimate errors in the memory-based drawing of the cat, the first 18 frontal view images of cats using a Google search for ‘cat’s head’ were measured and the mean eye level ratio was calculated using the same formula as described in Experiment 1 (mean ratio = .50). In the observational drawing task, we compared each participant’s drawn ratio with the ratio from the stimulus (.49).

##### Calculation of drawing errors of house

Here we were interested in the placement of the upper windows in relation to the height of the house, and we divided the distance between the bottom of the house and the top of the upper windows by the total height of the house. Due to the large variability in window placement on real houses, errors in window placement could not be accurately determined for the memory-based drawings. For the observational drawing, the ratio derived from the to-be-depicted image (.51) was compared to the ratio derived from each participant’s drawing.

##### Drawing questionnaire

This was the same as described in Experiment 1.

### Results and Discussion

#### Memory-based drawing of a cat

**Figure 4.**
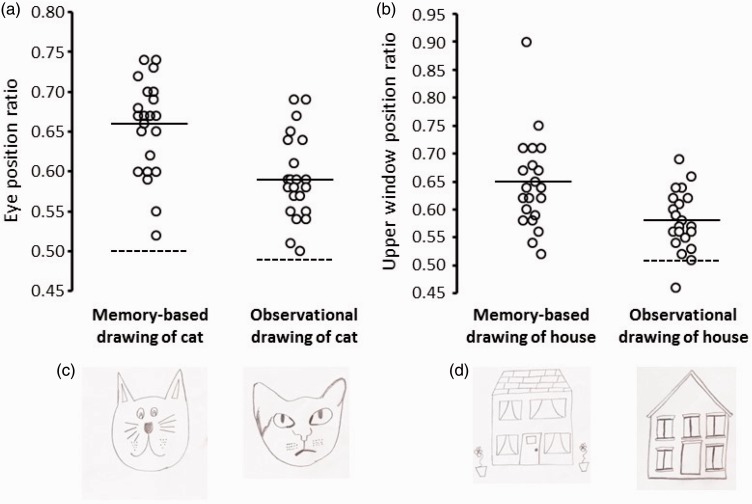
(a) Eye position ratios are plotted for each participant in the memory-based drawing and the observational drawing of a cat’s face. For the memory-based task, the dashed horizontal line shows the estimated mean eye position ratio for cat’s faces. For the observational task, the dashed horizontal line indicates the eye position ratio of the to-be-drawn cat. In both plots, the solid horizontal line displays the average eye position ratio of the drawn faces. (b) Each participants’ upper window position ratios are plotted for the memory-based drawing and the observational drawing of a house. In the memory-based task, no average upper window position ratio could be determined; therefore, there is no dashed horizontal line. In the observational task, the dashed horizontal line shows the ratio from the to-be-depicted house. In both plots, the solid horizontal line displays the mean upper window position ratio of the depicted houses. c) For illustrative purposes, drawings of cats produced by one participant. (d) One participant’s drawings of houses. All pictures are reproduced with the written permission of the participant.

Eye position ratios for each participant are plotted in Figure 4(a)*,* and one participant’s memory-based drawing of a cat is shown in Figure 4(c). A one sample *t* test comparing the mean value of the eye position ratio determined from the participants’ drawings with the mean estimated eye level ratio (from a Google search) revealed a significant difference, *t*(21) = 12.17, *p* < .001, Cohen’s *d* = 2.67, where the ratio was higher for participants’ drawings (*M* = .66, *SD* = .06) compared to the estimated value (*M* = .50).

#### Observation-based drawing of a cat

Individual eye position ratios for observational drawings are displayed in Figure 4(a). One participant’s drawing of the cat is displayed in Figure 4(c). A one sample *t* test revealed that the eye position ratio of the participants’ drawings was higher (*M* = .59, *SD* = .05) than the eye position ratio of the to-be-drawn cat (*M* = .49), *t*(21) = 9.03, *p* < .001, Cohen’s *d* = 2.0.

#### Relationship between errors for memory-based and observational drawings of cat

A Pearson’s correlation analysis revealed a significant positive relationship between eye position errors in the two tasks (*r* = .619, *p* = .002).

#### Memory-based drawing of a house

Individual upper window position ratios are displayed in Figure 4(b). An example of a participant’s memory-based drawing of a house is shown in Figure 4(d). The mean ratio of the placement of the upper windows for the memory-based drawing of a house was .65 (*SD* = .08).

#### Observation-based drawing of a house

Upper window position ratios are plotted in Figure 4(b), and one participant’s drawing of the house is displayed in Figure 4(d). A one sample *t* test revealed that the upper window position ratio of the participants’ drawings was higher (*M* = .58, *SD* = .05) than the window position ratio of the to-be-drawn image (*M* = .51), *t*(20) = 5.68, *p* < .001, Cohen’s *d* = 1.17.

#### Relationship of positioning between memory-based and observational drawings of house

A Pearson’s correlation analysis revealed a significant relationship between the upper window position ratios in the two tasks (*r* = .524, *p* = .015).

#### Relative area analysis

Using the same analyses as described in Experiment 1, we assessed whether the cat’s eyes were drawn larger (relative to the size of the head), and whether the windows were depicted larger (relative to the size of the house), compared to their size in the reference images. We found that the relative area occupied by the cat’s eyes in the drawings (*M* = 4.18%, *SD* = 1.32) was significantly larger than the relative area of the cat’s eyes in the to-be-depicted image (*M* = 3.11%), *t*(21) = 3.820, *p* = .001, Cohen’s *d* = .81. Further, results showed that the total relative area occupied by the windows in drawings of the house (*M* = 18.69%, *SD* = 5.05) was significantly larger than the total relative area of the windows in the reference image (*M* = 13.66%), *t*(20) = 4.565, *p* < .001, Cohen’s *d* = 1.0.

We found no correlation between errors in relative cat’s eye size (difference between drawn eye area vs. reference photo) and vertical eye position error (*r* = .279, *p* = .208). Further, we observed no association between errors in relative window size (difference between drawn window area vs. reference image) and vertical window position error (*r* = .279, *p* = .208).

#### Additional analyses

A linear regression analysis revealed that the magnitude of error in the observational drawing of the cat did not predict the degree of error in the observational drawing of the house (*r* = .025, *p* = .912).

We also tested (using a one-way ANOVA) whether the observational drawing ratio lay between the actual eye height ratio and the LTM ratio. For observational drawings of the cat, we found a significant difference between the ratios, *F* (2, 42) = 91.07, *p* < .001, and post-hoc *t* tests showed that the observational drawing ratio was significantly lower than the LTM ratio ( *p* < .001) but was higher than the actual ratio (*p* < .001). For house drawings, the same pattern of results was observed, *F*(2,40) = 37.54, *p* < .001, and post-hoc *t* tests showed that the observational drawing ratio was significantly lower than the LTM ratio ( *p* < .001) but was higher than the actual ratio ( *p* < .001).

In summary (see Table 1), Experiment 2 showed that systematic positioning of the eyes too far up the head in drawings of cats could not be explained by the ‘face-from-below’, the ‘hair-as-hat’, or the ‘head-as-box’ explanations. Additionally, Experiment 2 revealed that an upward positioning bias is also generalisable to drawings of non-face stimuli.

**Table 1.** Summary of Main Results.

Expt.	Task	Object	Mean ratio (*SD*)	Mean deviation from average/model	*t* (*df*)	*p*	Effect size (*d*)
#1	Memory	Human face	.57 (.06)	.09	7.35 (26)	<.001	1.55
#1	Obs.	Human face	.54 (.06)	.06	4.96 (26)	<.001	1.24
#2	Memory	Cat face	.66 (.06)	.16	12.17 (21)	<.001	2.67
#2	Obs.	Cat face	.59 (.05)	.10	9.03 (21)	<.001	2.00
#2	Memory	House	.65 (.08)	N/A^a^	N/A	N/A	N/A
#2	Obs.	House	.58 (.05)	.07	5.68 (20)	<.001	1.17

*Note*. Expt. = Experiment; Obs. = Observational.

a As average window placement could not be accurately determined for real houses, statistical analysis was not performed for this task.

## General Discussion

In Experiment 1, we replicated the results of previous studies demonstrating that non-artists draw the eyes too far up the head (Carbon & Wirth, 2014; Ostrofsky, 2015; Ostrofsky et al., 2014) and showed that the degree of positional error of the eyes in observational drawings was associated with the degree of error in memory-based drawings (Ostrofsky, 2015). Experiment 2 provided new insights into the cause of the bias, by showing that three previously proposed explanations (the ‘face-from-below’, the ‘hair-as-hat’, and the ‘head-as-box’ explanations (Carbon & Wirth, 2014)) could not fully explain the errors. Experiment 2 also showed that the upward positional bias was generalisable to non-face objects.

According to the ‘face-from-below’ explanation (Carbon & Wirth, 2014), the perspective from which children typically view human faces (i.e., from below) is proposed as a reason for the distorted spatial placement of the eyes in adults’ drawings of faces. Here we showed that participants drew the eyes too far up the head in memory-based and observational drawings of a cat’s head (typically viewed from above). This provides evidence that canonical representations of spatial relationships between features in faces are unlikely to be wholly derived from the perspective from which they were viewed as children. The distorted positioning of the eyes in drawings of cats may further suggest that the nature of the representations of faces stored in LTM is unlikely to consist of declarative knowledge (such as rules about eye placement derived from artistic manuals), given that rules for accurately drawing cat’s faces are presumably not widely known.

The tendency to position the eyes too far up the head when drawing a cat’s face could not be explained by the ‘hair-as-hat’ explanation, which assumes that the reason for the incorrect positioning of the eyes is that participants ignore the hair in their estimation of the height of the head, thus drawing the top of the head too low. Unlike a human’s head, a cat’s head lacks a distinct band of hair at the top of the head (see Figure 3(a)); therefore, this aspect could not explain the failure to position the eyes correctly. It should be noted, however, that due to differences in the distribution of hair on cats’ heads and human heads, the current results cannot falsify the ‘hair-as-hat’ explanation for eye position errors in drawings of human heads. Finally, the ‘head-as-box’ explanation could not explain the error in eye positioning in drawings of the cat’s face, as this assumes that eye positioning errors are caused by a failure to take into account the convexity of the top of the head, leading to an underestimation of the height of the head. In contrast to the convex shape of the top of a human’s head, the top of the cat’s head is concave, due to the outline of the ears (see Figure 3(a)), suggesting that positioning the eyes too high up could not be fully explained by the head-as-box proposal, at least for cat’s faces.

If the systematic bias to position the eyes too far up the head cannot be attributed to either visual perspective experiences from early life, the ‘hair-as-hat’, or the ‘head-as-box’ explanations, and the upward bias is generalisable to non-face objects, then where does the bias originate? A plausible explanation may be found in accounts of drawing errors that emphasise the importance of visual attention strategies in producing realistic drawings (Sutton & Rose, 1998). It is well known that eye fixations are concentrated on the most relevant features of a scene, such as people’s faces (Yarbus, 1967), and that observers make more eye movements to objects and regions that are judged to be informative within a scene (Loftus & Mackworth, 1978; Mackworth & Morandi, 1967). A credible suggestion is that attention is attracted to the most salient features or regions of an object and these features are then allocated a greater proportion of space within the outline of the drawn object compared to the less salient features. Indeed, for faces it has been shown that the majority of fixations occur on the eyes, nose, and mouth, which are located in the lower half of the head (Guo, Tunnicliffe, & Roebuck, 2010; Heisz & Shore, 2008), and importantly, the same pattern of fixations on these features has been observed during viewing of cats faces (Guo et al., 2010). For the observational drawing of a house (Figure 3(b)), the core features (i.e., features necessary to define its identity) were the windows, door, and roof (Picard & Vinter, 2005), two of which (i.e., windows and doors) were located in the bottom half of the picture. Thus, according to a feature salience account, if the majority of the core features are located in the bottom half of the image, relatively more space should be occupied by this part of the image in a drawing.

To provide support for the saliency account, we carried out an exploratory analysis of the relative area occupied by the eyes (and windows in the drawing of the house), in relation to the size of the head (house), and found that the eyes and windows were depicted larger in the drawings compared to the reference images. In fact, in the observational drawing of a face task in Experiment 1, the eyes in participants’ drawings occupied over twice as much relative area compared to those in the reference photos (2.16% vs. 1.05%). However, we found no association between the extent of the enlargement of the eyes and errors in the vertical positioning of the eyes, both for the human and cat face tasks. Similarly, there was no correlation between the drawn size of the windows and positional errors of the upper windows. These findings suggest that it is not the size of the depiction of individual salient features that leads to vertical position errors, but rather that vertical positioning errors may stem from an inaccurate depiction of relative size at a regional level. For example, several participants drew the windows approximately the correct relative size, but the upper windows were nevertheless positioned too high up because the negative space between the upper and lower windows was too large.

Future experiments could test the saliency account by manipulating the position of salient features within an object and assessing their placement in the drawing. Alternatively, the salience of different features within an image could be determined using eye-tracking technology, and the association with the accuracy of feature placement in a subsequent drawing could be analysed. Explicit instructions or methods to shift attention to different regions of the face (‘attentional priming’) could also be used to assess influences on vertical eye-drawing errors, as it has been shown that basic instructions to increase attention to the model led to more realistic drawings, at least for children (Sutton & Rose, 1998).

Interestingly, we found no association between the magnitude of errors in the cat’s face drawing task and the size of errors in the house drawing task. This suggests that the tendency to place features too high is object-specific, rather than consistent across object categories. Future research could test the extent to which participants show consistency in positional errors in observational drawings of several objects within a single category (e.g., drawings of different faces). Further, we found no evidence for a reduction in drawing errors by those participants who had previously received formal training in drawing. A possible explanation is that the self-reported training may have been too basic or taken place several years prior to the experiment. Finally, it should be noted that although the faces to be drawn in Experiment 1 were facing forward, they were not perfectly frontally aligned (see Figure 1). It is unlikely that the small deviation from exact frontal alignment affected the current results, but future studies could test whether viewing angle influences eye position errors, for instance whether similar errors would be observed when drawing faces in a profile view.
